# Esophageal perforation etiology, outcome, and the role of surgical management – an 18-year experience of surgical cases in a referral center

**DOI:** 10.1186/s12893-023-02080-w

**Published:** 2023-06-27

**Authors:** Reza Shahriarirad, Mohamadreza Karoobi, Ramin Shekouhi, Kamyar Ebrahimi, Keivan Ranjbar, Armin Amirian, Parviz Mardani, Mohammad Javad Fallahi, Bizhan Ziaian

**Affiliations:** 1 Thoracic and Vascular Surgery Research Center, Shiraz University of Medical Science, Shiraz, Iran; 2 School of Medicine, Shiraz University of Medical Sciences, Shiraz, Iran; 3 Department of Surgery, Shiraz University of Medical Sciences, Shiraz, Iran; 4 Department of Internal Medicine, Shiraz University of Medical Sciences, Shiraz, Iran

**Keywords:** Esophageal rupture, Esophageal perforation, Surgical management

## Abstract

**Introduction:**

Esophageal perforation is a surgical emergency with a high rate of morbidity and mortality. Its poor prognosis is mainly associated with previous patient-specific comorbidities and a lack of timely diagnosis and treatment. The objective of this study was to investigate the etiological factors and different surgical methods of treatment with consideration of mortality rate and comorbidities.

**Method:**

The present cross-sectional study was conducted on patients who underwent surgical intervention due to esophageal injury from 2002 to 2019 (18 years). Demographic and clinical characteristics along with performed surgical interventions were evaluated accordingly.

**Results:**

In this study, 69 patients with a mean age of 38.8 years were evaluated, of which 45 (65.2%) cases were men. In terms of location of the perforation, the thoracic portion of the esophagus followed by the cervical and abdominal esophagus were more frequently injured with a rate of 32 (46.4%), 30 (43.5%), and 19 (27.5%) cases, respectively. Accordingly, foreign body ingestion followed by penetrating injuries were the most common causative agents leading to esophageal perforation.

**Conclusion:**

Obtaining the desired results from the treatment of this condition depends on factors such as patients’ previous comorbidities, cause of the rupture, the location of the esophageal damage, and delay in the start of treatment. Since there is no single gold standard treatment strategy, each patient should be individually evaluated.

## Introduction

Esophageal Perforation (EP) is considered an uncommon yet life-threatening emergency associated with mortality rates of over 40% [1]. The high mortality rate of EP is mainly attributed to the relatively inaccessible location of the esophagus, followed by its proximity to vital organs. Furthermore, lack of durable serosa and collateral blood supply is the main pathogenesis posing the risk for this unique context [2]. EP is often divided into two main categories intraluminal and extraluminal. The factors associated with intraluminal esophageal injuries are mostly iatrogenic including endoscopy-related procedures, endoesophageal tube, and endotracheal intubation. Also, non-iatrogenic causative factors (e.g., barotrauma, caustic injury, and foreign bodies) could predispose patients to EP and its complications. the extraluminal esophageal injuries are mainly caused by blunt and penetrating injuries to the chest, and operation traumas [3–5].

Initial symptoms of EP are obscure and varied based on the location of the injury, and the causative agent. Unless it is associated with complications such as pneumothorax, sepsis, and shock. Accordingly, the most common symptoms presented initially are pain, fever, subcutaneous emphysema, and shortness of breath [6]. However, most symptoms of esophageal injury are non-specific. Thus, a high clinical suspicion and experience are important to confirm the diagnosis. The overall variety of non-significant clinical signs and symptoms combined with the lack of individual experience concerning this particular condition can hinder the rapid recognition of the potentially dangerous situation [7].

The treatment strategies rely mainly on the cause of the perforation, as well as the time from initiation of symptoms to hospital admission, and previous patient-associated comorbidities [8]. Although advanced diagnostic techniques have made a major impact in many areas of modern clinical practice, the diagnosis of EPs is daunting and can pose life-threatening complications [9]. Different procedures and therapeutic strategies have been introduced for EP including primary esophageal repair, simple drainage of the thoracic cavity, diversion esophagectomy, stenting of the perforation with a prosthesis, and esophageal resection with or without primary reconstruction [3, 8, 10]. However, a single goal standard treatment strategy to overcome this life-threatening condition is not yet clear. The objective of this study was to investigate the etiological factors and different surgical methods of treatment with consideration of mortality rate and comorbidities. Also, we aimed to provide surgeons with an extensive analysis of contributable factors as well as patient characteristics in this specific entity.

## Method and material

The present cross-sectional study was conducted on patients who underwent surgical intervention due to esophageal injury for a period of 18 years (2002–2019). Data was gathered from the main surgical referral center (Namazi hospital) affiliated with Shiraz University of medical science, in Shiraz, Iran. Inclusion criteria were all patients with the diagnosis of esophageal rupture or perforation who underwent surgical intervention.

Characteristics evaluated in the study include age, sex, the time interval between diagnosis and treatment, ASA score, location of rupture or perforation, etiology of perforation, surgical approach, surgical procedure, complication, and mortality rate with a follow-up duration of 90 days. Statistical analysis was performed using the SPSS software version 23, and by utilizing descriptive statistics, Chi-Square test, independent sample t-test, and linear regression. The level of significance was set at 0.05.

## Results

The patients who underwent surgery due to EP were enrolled in this research from 2002 to 2020. In this study, during the 18 years, a total of 69 patients were evaluated, demonstrating an average annual rate of 3.8 patients. The average age of the patients was 38.8 ± 19.5 years (range: 3 to 82 years) and 45 (65.2%) cases were men. Table 1 demonstrates the baseline features of patients with esophageal injury in our study.

**Table 1:** Demographic and clinical features of patients with esophageal injury in Shiraz, Iran from 2002–2019

Variable		Value
Age (year); mean ± SD		38.8 ± 19.5
Age group (year); n (%)	≤ 18	11 (15.9)
	19–25	8 (11.6)
	26–45	22 (31.9)
	46–65	21 (30.4)
	> 65	7 (10.1)
Year; n (%)	2002–2007	17 (24.6)
	2008–2013	15 (21.7)
	2014–2019	37 (53.6)
Season; n (%)	Spring	21 (30.4)
	Summer	19 (27.5)
	Winter	15 (21.7)
	Autumn	14 (20.3)
ASA score; n (%)	I	32 (46.4)
	II	25 (36.2)
	III	9 (13.0)
	IV	3 (4.3)
Symptom till hospitalization interval (hours)	Total; Median [Q1 – Q3]	40 [24–72]
	≤ 24	25 (36.2)
	> 24	47 (63.8)
Etiology; n (%)	Penetrating trauma	11 (15.9)
	Ingestion of caustic substance	7 (10.1)
	Foreign body ingestion	32 (46.4)
	Iatrogenic	10 (14.5)
	Underlying esophagus disease	9 (13.0)
	Infective Mediastinitis	17 (24.6)
	Spontaneous	1 (1.4)
Location	Cervical	30 (43.5)
	Thoracic	32 (46.4)
	Abdominal	19 (27.5)
Clinical Features	Chills and Fever	8 (11.6)
	Emphysema	10 (14.5)
	Dysphagia	5 (7.2)
	Tachycardia	3 (4.3)
	Tachypnea	1 (1.4)
	Chest pain	2 (2.9)
	Neck pain	1 (1.4)
	Dyspnea	1 (1.4)
Complication; n (%)	Mortality	3 (4.2)

The median duration of occurrence till diagnosis and hospitalization in the patients in our study was 40 h. None of the factors in our study had a significant association with the duration of hospitalization. The ASA score was calculated for each patient before surgical intervention, which is demonstrated in Table 1.

The surgical features of the patients in our study are demonstrated in Table 2. Secondary surgical repair was indicated for 11 (15.9) patients with signs of secondary EP following the first operation.

**Table 2:** Surgical features of patients with esophageal injury

Variables		Total	Age group (years)					Gender		ASA Score			
			≤ 18	19–25	26–45	46–65	> 65	Male	Female	I	II	III	IV
Total; n (%)		69 (100)	11 (17.5)	8 (11.1)	21 (29.2)	21 (29.2)	7 (9.7)	48 (66.7)	24 (33.3)	33 (45.8)	25 (34.7)	11 (15.3)	3 (4.2)
Surgical repair; n (%)	Primary	63 (91.3)	11 (17.2)	8 (12.7)	21 (33.3)	18 (28.6)	5 (7.8)	43 (68.3)	20 (31.7)	31 (49.2)	23 (36.5)	7 (11.1)	2 (3.2)
	Secondary	11 (15.9)	1 (9.1)	1 (9.1)	2 (18.2)	4 (36.4)	3 (27.3)	4 (36.4)	7 (63.6)	3 (27.3)	4 (36.4)	3 (27.3)	1 (9.1)
Type of Surgery; n (%)	Esophagectomy	26 (37.7)	1 (3.8)	2 (7.7)	10 (38.5)	9 (34.6)	4 (15.4)	14 (53.8)	12 (46.2)	8 (30.8)	14 (53.8)	3 (11.5)	1 (3.8)
	Thoracotomy	35 (50.7)	6 (17.1)	1 (2.9)	10 (28.6)	13 (37.1)	5 (14.3)	23 (65.7)	12 (34.3)	11 (31.4)	15 (42.9)	6 (17.1)	3 (8.6)
	Laparotomy	14 (20.3)	0 (0)	0 (0)	6 (42.9)	4 (28.6)	4 (28.6)	10 (71.4)	4 (28.6)	4 (28.6)	7 (50.0)	3 (21.4)	0 (0)
	jejunostomy tube insertion	23 (33.3)	1 (4.3)	3 (13.0)	8 (34.8)	8 (34.8)	3 (13.0)	15 (65.2)	8 (34.8)	7 (30.4)	13 (56.5)	2 (8.7)	1 (4.3)
	Gastrostomy Tube Insertion	21 (30.4)	2 (9.5)	0 (0)	8 (42.9)	6 (28.6)	4 (19.0)	16 (76.2)	5 (23.8)	7 (33.3)	10 (47.6)	3 (14.3)	1 (4.8)
	Chest Tube Insertion	29 (42.0)	2 (6.9)	1 (3.4)	9 (31.0)	12 (41.4)	5 (17.2)	18 (62.1)	11 (37.9)	7 (24.1)	15 (51.7)	6 (20.7)	1 (3.4)
	Neck Exploration Surgery	25 (36.2)	3 (12.0)	5 (20.0)	5 (20.0)	9 (36.0)	3 (12.0)	18 (72.0)	7 (28.0)	11 (44.0)	8 (32.0)	5 (20.0)	1 (4.0)
	Explorative Laparotomy	8 (11.6)	0 (0)	2 (25.0)	3 (37.5)	3 (37.5)	0 (0)	5 (62.5)	3 (37.5)	3 (37.5)	4 (50.0)	0 (0)	1 (12.5)
	Esophagectomy	66 (95.7)	10 (15.2)	7 (10.6)	22 (33.3)	20 (30.3)	7 (10.6)	43 (65.2)	23 (34.8)	31 (47.0)	24 (36.4)	9 (13.6)	2 (3.0)
	Jejunectomy	16 (23.2)	1 (6.3)	4 (25.0)	1 (6.3)	9 (56.3)	1 (6.3)	10 (62.5)	6 (37.5)	6 (37.5)	8 (50.0)	2 (12.5)	0 (0)
	Tracheotomy	2 (2.9)	0 (0)	1 (50.0)	0 (0)	0 (0)	1 (50.0)	2 (10)	0 (0)	1 (50.0)	0 (0)	1 (50.0)	0 (0)

In this study, surgical treatment methods for patients with esophageal rupture are shown in Table 2. Accordingly, the surgical method used in our study were divided into two groups primary and secondary repair, in which 13 (18.1%) patients underwent a second surgery following complications or surgical site infection. There was no significant association between primary and secondary surgery, and the patients age, gender, or ASA scores.

The most common surgical method used in our study was esophageal repair with primary suture, which was used in half of the patients, and in 2 cases this repair was amplified by a flap. Also, esophagectomy was required in 26 (37.7%) patients. That said, trans-Hiatal esophagectomy was the least used method for esophageal repair and was performed in only 4 (5.8%) cases.

According to Tables 2, 29 (42.0%) of patients underwent chest tube insertion. Interestingly, our study revealed that chest tube insertion was performed more frequently on patients with ASA scores II and III, and also higher age groups, which was also statistically significant (P = 0.07 and 0.02, respectively). Furthermore, patients with higher ASA scores more frequently underwent thoracotomy (P = 0.04). Also, laparotomy had a significant association with higher age groups (P = 0.04).

In this study, 3 (4.3%) patients passed away, one was a 4-year-old girl with caustic substance ingestion with damage to the thoracic and abdominal esophagus (ASA 4). Another case was a 63-year-old male with foreign body ingestion and mediastinitis with a para-esophageal abscess in the abdominal esophageal portion (ASA 4). The last case was a 41-year-old male with complaint of chronic dysphagia and weight loss, in which imaging studies demonstrated distal esophageal perforation with periesophageal abscess formation with ASA of 1. There was no significant relationship between the type of surgical intervention and mortality.

Table 3 demonstrates the etiology and location of EP. As demonstrated, foreign body ingestion followed by penetrating injuries were the most common causative agents leading to EP. In terms of location of the perforation, the thoracic portion of the esophagus followed by the cervical and abdominal esophagus were more frequently injured with a rate of 32 (46.4%), 30 (43.5%), and 19 (27.5%) cases, respectively (Table 3). Accordingly, 12 (17.3%) patients had a perforation in more than one portion of the esophagus. It is worth mentioning that a number of patients had EP in multiple areas, such as five cases with thoracic and abdominal EP, five cases of cervical and thoracic EP; one case of cervical and abdominal EP (Bone FB ingestion), and one with cervical, thoracic, and abdominal EP due to FB (bone) ingestion in a 59-year-old male.

**Table 3:** Etiology of esophageal perforation patients based on the location of injury and age

Etiology	Total; *N = 69*	Location			Age group (years)				
		Cervical	Thoracic	Abdominal	1–18	19–25	26–45	46–65	> 65
Foreign body ingestion	32 (46.4)	16 (53.3)	15 (46.9)	8 (42.1)	2 (18.2)	1 (12.5)	12 (54.5)	14 (66.7)	3 (42.9)
Penetrating trauma	11 (15.9)	7 (23.3)	4 (12.5)	0 (0)	3 (27.3)	5 (62.5)	3 (13.6)	0 (0)	0 (0)
Iatrogenic	11 (14.5)	3 (10.0)	4 (12.5)	4 (21.1)	3 (27.3)	0 (0)	2 (9.1)	1 (4.8)	4 (57.1)
Caustic injury	7 (10.1)	3 (10)	5 (15.6)	2 (10.5)	1 (9.1)	1 (12.5)	4 (18.2)	1 (4.8)	0 (0)
Spontaneous	1 (1.4)	0 (0)	1 (3.1)	0 (0)	0 (0)	0 (0)	0 (0)	1 (4.8)	0 (0)

Based on statistical analysis, penetrating trauma was significantly more less in abdominal EP (P = 0.03). Furthermore, age was significantly associated with foreign body ingestion (P = 0.02), penetrating trauma (P < 0.001), and iatrogenic (P = 0.01) etiologies. The average age of patients with foreign body ingestion and penetrating trauma etiologies were significantly higher, while iatrogenic EP was more frequently seen among the youngest and also oldest age groups.

## Discussion

EP is a surgical emergency with a high rate of morbidity and mortality [11]. Unfortunately, the prevalence of EP has been increasing dramatically in the last decades [12]. In this study, we aimed to report our 18-year experience with this rare, life-threatening condition. We demonstrated that EP is more prevalent amongst men compared to women. Also, our study introduced foreign body ingestion, as the main etiology of EP. According to our study, the most common cause of EP was foreign body ingestion, followed by trauma, and iatrogenic injuries. EPs caused by foreign body ingestion most often affect the cervical esophagus, while spontaneous and iatrogenic ruptures are more common in the distal segment [13, 14].

The main reason for this finding is the presence of anatomical narrowing of the esophagus at the cricopharynx, aortic arch, and the gastroesophageal junction, which most often leads to obstruction by foreign bodies and subsequent wall necrosis and rupture [15]. Also, our study showed that penetrating injuries are mainly associated with damage to the cervical esophagus. Contrary to our results, previous studies suggested that traumatic EP mainly injures the distal third of the esophagus, and is a life-threatening cause of esophageal rupture, especially penetrating injuries from bullets or stab wounds that are frequently associated with extensive damage to other vital organs [16]. Iatrogenic injuries to the esophagus were the 3rd main cause of EP in our study. Interventions using therapeutic endoscopies, such as pneumatic dilation, stent placement, foreign body removal, and endoscopic ablation techniques can significantly increase the risk of perforation [17].

Regarding the location of esophageal rupture, our study reported that thoracic EP (n = 32; 46.4%), followed by abdominal (n = 30; 43.5%), and cervical esophagus (n = 19; 27.5%) were most commonly injured locations, respectively. On the contrary, various reports have suggested the cervical esophagus to be the most common site of EP [9, 15, 16]. Fortunately, cervical esophageal ruptures are associated with less mortality than thoracic and abdominal EP which is due to limited spreading of infection to the mediastinum, pleura, and better accessibility for treatment [13].

Early diagnosis and treatment are essential to achieve optimal results and reduce mortality. Although direct x-ray and contrast-associated tomography could provide important findings for the diagnosis of EP initially, it could be misleading [18]. Diagnostic endoscopy, which is performed almost exclusively with flexible endoscopes (FE), provides direct visualization of the esophagus that could be used in the closure of small mucosal defects associated with EP. However, the usage of esophagoscopy in the diagnosis of EP is controversial. Despite the low risk of perforation, the availability of other modalities has always questioned the indications for performing FE. Operate dependence, contamination of the ruptured esophagus increased chance of sepsis, and extending the perforation due to mucosal manipulation by endoscopes are considered significant downfalls of esophagoscopy [19].

To date, many treatments have been tried, but a single gold standard treatment option is still unclear. The main goals in the treatment of esophageal rupture are hemodynamic stabilization, repair of the damaged wall, prevent further extraluminal contamination, and antibiotic therapy [20]. Thus, choosing the best treatment strategy for patients should be individually discussed. Several factors are associated with the selection of the type of therapeutic intervention, including time of diagnosis(most influential factor), the cause of injury, the primary location of the EP, age, and clinical comorbidities of the patient [21].

Overall, three main treatment options exist for better management of EP. First, non-surgical interventions including conservative treatments to improve patients’ nutritional status, as well as antibiotic therapy for sepsis prevention [22]. Also, minimally invasive techniques including endoscopic treatments are being used increasingly in the last decades [23]. However, endoscopic interventions remain for patients with stable hemodynamic status without any signs of sepsis, who were diagnosed early. Endoscopic clips and stenting are the most common endoscopic interventions used for the repair of the damaged esophageal wall.

Surgery plays a major role in the treatment of EP. It provides sufficient esophageal rupture closure and allows the removal of esophageal contents from the thoracic cavity. These interventions include drainage with or without decortication, primary esophageal repair, esophagectomy, or esophageal exclusion [24]. The selection of surgical intervention mainly depends on the patient’s hemodynamic status, previous comorbidities, and the extent of esophageal damage. Accordingly, initial repair with or without augmentation is probably the standard treatment for EF in most centers. However, few studies suggest that initial repair should only remain for patients with primary perforations, and recommend resection or diversion when the perforation is longer than 24 h [25].

The most common surgical method used in our study was primary esophageal repair (91.3%) compared with secondary repair (15.9%). Also, the primary suture was performed for half of patients with EP with 2 cases this repair was amplified by a flap. However, primary surgical repair may not always be effective especially if signs of advanced mediastinitis or infection were observed, in which case discontinuity resections are a better choice for further surgery [26]. Our previous report has demonstrated the higher efficacy of continuous sutures in reconstructive surgery [27]. More aggressive surgical methods including esophageal resection, diversion, exclusion, or T-tube insertion could be considered for patients with severe esophageal injury and simultaneous infection [28]. Also, based on the location of EP, concomitant damage to the adjacent organs should always be considered. In our study, 16 patients underwent jejunum reconstruction due to the severity of the damage.

Surgical Treatment for cervical esophageal rupture should be done within the first 16 to 24 h following the injury. Previous studies suggest that conservative treatment with antibiotics and a nasogastric tube may be helpful in small ruptures less than 2 cm [29]. However, primary repair and drainage remain an old standard surgical technique for large EPs. Cervical esophagostomy with gastrostomy or jejunostomy is mainly used for patients with extensive mediastinitis, signs of esophageal necrosis or obstruction, hemodynamic instability, and patients who are unable to tolerate surgical repair or resection [30].

An abdominal puncture should be performed with a midline laparotomy. After debridement of necrotic tissue, single- or double-layer tension-free closure of the hole should be performed. It is recommended to tighten the esophageal suture with gastroplasty using the gastric fundus (such as complete or partial fundoplication) and to insert the nasogastric tube, making a nourishing jejunostomy, and perform external drainage of the subphrenic space [18].

In our study, no significant association were observed between study variables and the rate of mortality. It can be speculated that the low death rate of our patients might be the reason that we did not find any significant relations between type of surgical interventions and the overall mortality rate. In addition, patients having foreign body ingestion are generally associated with more favorable outcomes when it comes to surgical repair of EP. Generally, one of the most important determinants of mortality is the time interval between the onset of symptoms and treatment. In a review of 726 cases of esophageal rupture, Brinster et al. reported that the mortality rate amongst patients that received treatment less than 24 h after the onset of symptoms, was only 14% [9, 16]. There was no significant association between the surgical approach and mortality in our study, which seems that it depends more on the characteristics of esophageal rupture and the general condition of the patient than on the surgical method. Among the limitations of this study, is the lack of a long-term follow-up period for patients with EP. Also, based on the retrospective nature of our study, we were unable to obtain all of the patients’ information, especially regarding their clinical presentations, due to incomplete hospital records. Thus, due to the challenging nature of this disorder and the lack of a single therapeutic strategy, future studies are required to determine a specific surgical strategy to conclude the most effective therapeutic approach in the face of this condition.

## Conclusion

Although EP is associated with high morbidity and mortality, we achieved a low mortality rate with rapid diagnosis and management. Obtaining the desired results from the treatment of this condition depends on factors such as patients’ previous comorbidities, the cause of the rupture, the location of the esophageal damage, and delay in the start of treatment. Since there is no single gold standard treatment strategy, each patient should be individually discussed.

## Data Availability

The datasets used and/or analyzed during the current study are available from the corresponding author on reasonable request and with permission of the Research Ethics Committee of the School of Medicine-Shiraz University of Medical Sciences.

## Abbreviations

EP: Esophageal perforation
